# Warfarin dosing strategies evolution and its progress in the era of precision medicine, a narrative review

**DOI:** 10.1007/s11096-022-01386-8

**Published:** 2022-03-05

**Authors:** Amr Mohamed Fahmi, Hazem Elewa, Islam El Jilany

**Affiliations:** 1 Clinical pharmacist, Hamad Medical Corporation, Doha, Qatar; 2 College of Pharmacy, QU Health, Qatar University, P.O. Box 2713, Doha, Qatar; 3 Biomedical and Pharmaceutical Research Unit, QU Health, Qatar University, Doha, Qatar; 4 School of Pharmacy, University of Florida, Gainesville, USA

**Keywords:** CYP2C9, Genetic-guided warfarin dosing, Warfarin clinical dosing, Warfarin dosing algorithms, Warfarin fixed dosing, VKORC1—CYP4F2

## Abstract

*Background* For decades, vitamin K antagonists and specifically warfarin, have been the sole agents used orally to manage thromboembolic conditions, including stroke and venous thromboembolism (VTE). Several factors lead to warfarin dose variability, including genetic and non-genetic factors which made warfarin management challenging especially at the initiation phase. To overcome the challenges with warfarin dosing at initiation, strategies other than conventional or fixed dosing were introduced and explored. *Aim* In this narrative review, we aim to discuss and critique the different dosing strategies for warfarin at initiation with more focus on genotype-guided warfarin dosing and the most recent supporting evidence for and against its use. *Method* Medline database was searched from 1965 to July 2021. Articles addressing different warfarin dosing methods were screened for inclusion. *Results* A number of methods exist for warfarin initiation. Studies comparing different dosing methods for initiation yielded conflicting outcomes due to differences in study design, population studied, comparator, and outcomes measured. *Conclusions* Looking at the big picture, the use of genetic dosing for warfarin initiation can lead to better outcomes. Whether these better outcomes are clinically or economically beneficial remains controversial.

## Impacts on practice

- Warfarin remains the drug of choice for the prevention of thrombosis in a number of indications and therefore a focus on the best dosing methods is still needed.
- Genetic-guided warfarin dosing is the most precise method, but not the most widespread.
- We describe most common warfarin dosing methods which could help prescribers of vitamin k antagonists.
- We summarize the most common warfarin genetic variants and dosing algorithms and how they compare to empirical warfarin dosing.

## Introduction

For decades, vitamin K antagonists and specifically warfarin, have been the sole agents used orally to manage thromboembolic conditions, including stroke, deep vein thrombosis (DVT) and pulmonary embolism (PE). With the discovery of direct oral anticoagulants (DOACs), more healthcare providers prefer them over warfarin due to their ease of use, fast onset and offset, and the lack of need for monitoring. In the last decade in the United States, warfarin prescribing dropped from 77 to 12% in specific indications like atrial fibrillation (AF) [1]. Despite the increased use of DOACs, warfarin remains the main drug of choice for stroke prevention in patients with mechanical heart valve replacement, AF with significant mitral valve stenosis or hypertrophic cardiomyopathy, and antiphospholipid antibody syndrome [2, 3]. Warfarin is also preferred in obese patients (body mass index > 40 Kg/m^2^), those with poor renal function (creatinine clearance < 15 ml/min) and breastfeeding women [4].

Warfarin mediates its anticoagulant effect by inhibiting the vitamin K epoxide reductase enzyme (VKOR). The inhibition of VKOR leads to a decrease in the reduced (active) form of vitamin K, which inhibits the activation of the coagulation factors II, VII, IX, and X and produces the anticoagulant effect of warfarin [4]. Several factors lead to warfarin dose variability, including genetic and non-genetic factors [4]. Almost half of this interpatient variability has been attributed to clinical and genetic factors, while the remaining variability remains unexplained [5]. And since warfarin has a narrow therapeutic window, dosing variability has made warfarin management challenging especially at the initiation phase. It also makes the frequent measurement of international normalized ratio (INR) necessary to ensure optimal anticoagulation. To overcome the challenges with warfarin dosing at initiation, strategies other than conventional or fixed dosing were introduced and explored [6].

## Aim

In this narrative review, we discuss and critique the different dosing strategies for warfarin at initiation with more focus on genotype-guided warfarin dosing and the most recent supporting evidence for and against its use.

## Method

Medline database was searched from 1965 to July 2021 for keywords such as: warfarin, pharmacogenomics, pharmacogenetics, warfarin pharmacogenomics, warfarin dosing and warfarin algorithms. Search was limited to English language, humans, and adults (18 years and older). Related articles were also screened to capture any articles not reported in the results. The first and second investigators screened the resulting articles for inclusion. Articles were included if the authors addressed:

1. Warfarin clinical dosing: reported the significance of different clinical variables affecting warfarin dosing e.g.: Age, body surface area (BSA), sex, diet and medications.
2. Warfarin fixed dosing: compared different warfarin loading doses or warfarin nomograms at initiation.
3. Genetic-guided warfarin dosing: compared different warfarin pharmacogenetic dosing algorithms to each other or derived their own algorithm. Also, if pharmacogenetic-guided dosing was compared to clinical or fixed dosing.

## Results

### Fixed dosing

Fixed dosing is one of the most widely used methods for warfarin dosing. It involves administering a predetermined warfarin dose for two consecutive days and measuring the INR on the third or fourth day. Then, clinicians revisit a warfarin nomogram, which contains warfarin doses according to patient’s INR, and recommends dose modification according to the observed INR. Clinicians may prefer to give a loading dose, which is defined as “an initial warfarin dose” for 1–2 days [7]. A typical loading dose ranges from 5 to 10 mg [8].

In the last three decades, a number of trials were published looking at how the use of usual warfarin doses “≤ 5 mg” compare to the use of high warfarin doses “> 5 mg”. These trials had differences in their designs, outcomes and yielded conflicting results. In a small prospective study, Crowther et al. compared an initial dose of 5 mg versus 10 mg. Most of the patients had acute thromboembolism. There were no differences between the two groups in achieving a therapeutic INR on two consecutive days, i.e., days 3 and 4 or days 4 and 5 of treatment [9]. It should be noted that most of the patients in this study were hospitalized, which may have made them more sensitive to warfarin therapy when compared to those in the outpatient setting. This sensitivity can be attributed to other co-morbid diseases and poor dietary intake [10]. Kovacs et al. tested the 5 mg nomogram used by Crowther et al. in comparison to his 10 mg nomogram in outpatients suffering from VTE [10]. Patients in the 10 mg group achieved therapeutic INR 1.4 days earlier than the 5 mg nomogram without increasing the risk of bleeding. Additionally, patients in the 10 mg group were more likely to achieve a therapeutic INR by day 5 (83% vs. 46%; *P*-value (*P*) < 0.001). Although this study was one of the main studies advocating for a loading dose of 10 mg, it is important to note that the authors excluded patients with high bleeding risk. A third open-label randomized study by Quiroz et al. comparing two warfarin initiation nomograms (5 mg vs. 10 mg) found no difference in the time needed to achieve therapeutic INR for two consecutive days (median time = 5 days, log-rank test *P* = 0.69) [11]. Another retrospective cohort study included outpatients who were treated for VTE and were started on the 10 mg nomogram [12]. The aim was to increase the generalizability of the previously published 10 mg nomogram. Compared to patients who were non-adherent to the nomogram, those who were adherent were more likely to have a therapeutic INR by day 5, whereas no difference existed in bleeding and thrombosis recurrence. A systematic review and meta-analysis published in 2013 and updated in 2016 included all randomized trials comparing loading dose of 5 mg versus 10 mg. The authors concluded that there were no differences between the 5 mg and 10 mg groups in the outcome of INR in range by day 5 [Relative risk (RR) = 1.17, 95% CI 0.77–1.77, *P* = 0.46], although the heterogeneity was high (I^2^ = 83%) [13, 14]. During sensitivity analyses, removal of the study with the highest contribution to heterogeneity confirmed a lack of difference between the two groups (RR, 0.98; 95% CI, 0.73–1.32, *P* = 0.9, I^2^ = 44%).

The 2012 American College of Chest Physicians (ACCP) guidelines for oral anticoagulants recommend starting patients on a 10 mg warfarin loading, especially in healthy ambulatory individuals [8]. The guidelines were published before the previously mentioned meta-analysis, which may explain the discrepancies between the recommendation and the evidence from the meta-analysis. The previous 2008 ACCP guidelines and the British Society for standards of Hematology (BCSH) 2011 provide options to give patients a loading dose of 5–10 mg [15, 16].

In summary, the evidence is inconclusive to recommend 10 mg over 5 mg as a loading dose. Based on the literature, patients who were more likely to benefit from the 10 mg regimen were outpatients, younger, had higher vitamin K intake, less co-morbid diseases, and fewer drug interactions.

### Clinical dosing

Multiple factors can affect warfarin dosing, including genetic and non-genetic (clinical) factors, both of which explain more than half of the variability in warfarin dose requirements [17]. Apart from genetics, non-genetic factors were explored in several studies [17–19]. Some of the well-established clinical factors affecting warfarin are age, sex, body mass index, race, baseline INR, indication/target INR, liver disease, and drug-drug interactions.

Age is one of the earliest identified factors affecting warfarin dose [20]. Redwood et al. examined the effect of age on warfarin dose and concluded that, on average, patients greater than 70 years of age require 25–30% lower warfarin doses than those less than 30 years of age [21]. Similarly, a stud by Gurwitz et al. assessed the effect of aging on oral anticoagulant dose requirements [22]. Patients who were in the highest age group were more likely to require a lower warfarin dose. Also, those between 50 and 70 years of age had a 10% reduction in their mean warfarin dose as compared to the younger group (7.2 mg vs. 8.1 mg; *P* = 0.0002). In the sub-group of patients above 70 years of age, there was a higher incidence of bleeding when compared to those younger than 50 years of age (RR, 1.8; 95% CI, 1.12-3; *P* = 0.016) [23]. Gage et al. quantified the impact of age on warfarin dose, and he observed that every decade increase in age was associated with a 7–13% decrease in warfarin maintenance dose [18].

Weight and its surrogates BSA and body mass index (BMI) is another widely studied factor associated with warfarin dose variability. According to the Gage algorithm, with every 0.26 m^2^ increase in BSA, warfarin dose increased by 15% [18]. Another study showed that an increase in BMI by 1 point is associated with a 0.69 mg increase in weekly warfarin dose requirements [24]. Others have shown that BMI > 40 kg/m^2^ (morbidly obese) was significantly associated with higher warfarin dose requirements when compared with obese, overweight, normal weight, and underweight individuals [25].

Race has been an important source of variability for many drugs, including warfarin. This effect was highlighted in a study by Absher and colleagues, which included 146 patients from a community hospital and an outpatient anticoagulation clinic. African American race was one of 5 factors associated with a warfarin dose higher than 5 mg. Also, African American patients required extra 1.3 mg daily warfarin dose when compared to their white counterparts [26]. Another retrospective cohort of 345 patients found that African American patients had the highest average weekly warfarin doses [43 mg (39–47)], followed by Whites [36 mg (34–39) ], Hispanics [31 mg (25–37)], while the lowest average weekly warfarin doses were for those with Asian ancestry [24 mg (21–27)] [27].

Numerous drugs interact with warfarin, a good number of which can affect warfarin maintenance dose, especially when the interacting medication is started or stopped. Warfarin is a racemic mixture of S-warfarin and R-warfarin where the S-isomer is more potent than the R [8]. S-warfarin is primarily metabolized by Cytochrome P 450 2 C9 (CYP2C9), while R-warfarin is metabolized by Cytochrome P 450 3 A4 (CYP3A4). Warfarin drug interactions are either pharmacokinetic or pharmacodynamic interactions [8]. The pharmacokinetic interactions are mainly due to induction or inhibition of CYP2C9, which is the main enzyme responsible for the metabolism of warfarin (and/or CYP3A4 at a lesser extent) [8]. Patients on a stable maintenance dose of warfarin who are started on a potent drug interacting medication usually require a change in warfarin dose [8]. Most commonly, amiodarone, azole antifungals, phenytoin, rifampicin, trimethoprim/sulfamethoxazole, and the use of statins are included in warfarin maintenance dose algorithms. However, quantification of the effect of the interacting drug on warfarin dose is complicated and varies based on the interacting medication, genetic variants affecting the responsible metabolizing enzyme and other inter-individual variations [28]. For example, 30–50% warfarin dose reduction is recommended with amiodarone initiation, whereas a 25–33% reduction is recommended for fenofibrate [29].

Most of the demographic and clinical elements discussed above were incorporated in algorithms along with genetic factors and then introduced as genetic algorithms. However, these algorithms allow healthcare providers to use the clinical factors alone, especially when genetic factors are unknown or pending, to predict a warfarin maintenance dose, although with less accuracy. Those algorithms will be discussed in the genetic algorithms section.

Genotype-guided warfarin dosing

### Genotype-guided warfarin dosing

Warfarin dosing is affected by a number of genetic variants that could unease the process of initiation and discontinuation. The main three genes that carry these variants are *CYP2C9, VKORC1*, and *CYP4F2* (Table 1). *CYP2C9* codes for CYP2C9 which plays an important role in clearing the more potent S-warfarin enantiomer. The *VKORC1* on the other hand codes for VKOR enzyme, which is responsible for the activation of vitamin K, which in turn activates the coagulation factors. Warfarin exerts its anticoagulant effect through inhibition of the VKOR enzyme. The *CYP4F2* codes for CYP4F2 enzyme, which is responsible for metabolizing the activated form of vitamin K, rendering it inactive.

**Table 1:** Most prominent genetic variants affecting warfarin dose

Gene	*CYP2C9*	*VKORC1*	*CYP4F2*	*CYP2C*	*GGCX*	*CALU*
Location [41]	Chromosome 10	Chromosome 16	Chromosome 19	Chromosome 10	Chromosome 2	Chromosome 7
Function [6]	Encodes for CYP2C9 enzyme which metabolises the more active S-warfarin.	Encodes for VKOR enzyme which changes oxidized vitamin k into reduced (active) vitamin k, which activates the clotting factors.	Encodes for CYP4F2 enzyme which deactivates the reduced form of vitamin k.	Intergenic cluster, could affect *CYP2C9* gene activity.	Encodes for GGCX enzyme which activates the coagulation factors.	Encodes for CALU protein which chaperon GGCX enzyme.
Common variants and most prevalent race [6, 34]	CYP2C9*2 (rs1799853): Missense variant.: in Caucasians. CYP2C9*3 (rs1057910) Missense variant: in Caucasians. CYP2C9*5 (rs28371686) Missense variant: in Africans. CYP2C9*6 (rs9332131) Frameshift variant: in Africans. CYP2C9*8 (rs7900194) Missense variant: in Africans. CYP2C9*11 (rs28371685) Missense variant: in Africans.	VKORC1 c.-1639G > A upstream single nucleotide variant.: AA: common in Asian population AG: common in Caucasian population. GG: common in African population.	CYP4F2*3 (rs2108622)Missense variant: Most common in Asians and Caucasians and associated with a higher warfarin maintenance dose.	SNP (rs12777823) Most prevalent in Africans and associated with a lower warfarin maintenance dose.	Non-synonymous variant (rs11676382). Prevalent in Caucasian and associated with lower warfarin maintenance dose. [42]	Intronic SNP (rs339097) Associated with higher warfarin maintenance dose in Africans. [43]

CALU: Calumenin, CYP2C9: Cytochrome 450 2C9, CYP4F2*3: Cytochrome 450 4F2*3, GGCX: Gamma glutamyl carboxylase, SNP: Single nucleotide polymorphism, ,VKORC1: Vitamin k epoxide reductase complex 1

Ever since the FDA proposed the pharmacogenetic dosing table to be added to warfarin labeling [30], groups and consortia such as the Pharmacogenomics Knowledge Base (PharmGKB) and the Dutch Pharmacogenetics Working Group (DPWG) started publishing guidelines on how to use the available genetic information to optimize warfarin dosing [31, 32]. Among these resources are the clinical Pharmacogenetics Implementation Consortium “CPIC” guidelines, which help clinicians understand how genetic test results can be used in prescribing decisions for certain medications [33]. Both the American Society of Health-system Pharmacist “ASHP” and the American Society of Clinical Pharmacology and Therapeutics “ASCPT” endorse the CPIC guidelines. The CPIC guidelines for *CYP2C9* and *VKORC1* genotypes and warfarin dosing were published in 2011 and updated in 2017 [34].

### Warfarin genetic algorithms

Genetic dosing uses patients’ genetic test results to predict the initial warfarin dose. There are mainly two approaches to genetic dosing; the first is the FDA pharmacogenetic table, which can be found in the warfarin package insert and was added in early 2010 (Table 2) [35]. With the results of patients’ genetic testing for *CYP2C9* and *VKORC1* (*c.-1639G > A*), clinicians can use this table as a guide to recommend the most appropriate warfarin dose. The table generally accounts for clinical factors (e.g., age, weight, sex, race, concomitant drugs, and comorbidities) but doesn’t account for other genetic variants that may affect warfarin maintenance dose variability. The second approach is through the use of pharmacogenetic algorithms, which often include genetic components in addition to clinical factors. These pharmacogenetic algorithms are derived as linear regression equations from cohorts where every variable is tested for association with warfarin maintenance dose. Then, variables with the significant association are added to a model to be computed in the multiple linear regression analysis to yield a final equation with the highest degree of association (coefficient of determination, R^2^). The next step is the validation process which is done on a smaller similar population to compare the observed dose to the predicted dose using the algorithm. Once validated, some important measures are calculated, e.g., the mean absolute error, and the percentage of predicted warfarin doses within 20% of the actual warfarin maintenance dose. Several pharmacogenetic algorithms are available for different races. Some of these algorithms incorporate clinical factors in addition to genetic factors, which has led to a better dose prediction and refinement for warfarin maintenance doses. Of these, two algorithms are the most validated; Gage et al. and IWPC algorithms (Table 3) [17, 19]. Other algorithms can also assist in predicting the loading dose (Avery et al.) and to refine warfarin dose after the initiation phase (Lenzini et al.) [36, 37].

**Table 2:** Suggested warfarin dose based on *VKORC1/CYP2C9* genetic profile

*VKORC1\CYP2C9*	*1/*1	*1/*2	*1/*3	*2/*2	*2/*3	*3/*3
GG	5–7 mg	5–7 mg	3–4 mg	3–4 mg	3–4 mg	0.5–2 mg
GA	5–7 mg	3–4 mg	3–4 mg	3–4 mg	0.5–2 mg	0.5–2 mg
AA	3–4 mg	3–4 mg	0.5–2 mg	0.5–2 mg	0.5–2 mg	0.5–2 mg

(Pharmacogenetic table) Bristol-Myers Squibb. Coumadin (warfarin sodium) package insert

**Table 3:** Comparison of the Gage et al. and IWPC pharmacogenetic algorithms

	Gage et al.	IWPC
Date	Published 2008	Published 2009
Derivation cohort	1015 patients	4043 patients
Validation cohort	292 patients	1009 patients
Age	Derivation cohort: Mean: 57 years Validation cohort: Mean: 65 years	Validation cohort: 53.6% > 60 years Derivation cohort: 52.3% > 60 years
Genetic factors	CYP2C9*2 CYP2C9*3 *VKORC1 c.-1639 G > A*	CYP2C9*2 CYP2C9*3 *VKORC1 c.-1639 G > A*
Clinical factors	BSA, Age, Race, Sex, Amiodarone usage, Smoking status, Target INR and prior DVT/PE	Age, Height, Weight, Race, Enzyme inducer status and Amiodarone status
R^2^	54%	43%
MAE	7 mg/wk	8.5 mg/wk
Regression equation	$$\begin{gathered} \exp \left[ {0.9751{\text{ }} - {\text{ }}\left( {0.3238{\text{ }} \times VKORC1639G > A} \right){\text{ }} + {\text{ }}\left( {0.4317{\text{ }} \times ~BSA} \right)} \right. \hfill \\ \,\, - {\text{ }}\left( {0.4008{\text{ }} \times CYP2C9*3} \right){\text{ }} - {\text{ }}\left( {0.00745{\text{ }} \times {\text{ }}Age} \right){\text{ }} - {\text{ }}\left( {0.2066{\text{ }} \times CYP2C9*2} \right)~ \hfill \\ \,\, + {\text{ }}\left( {0.2029{\text{ }} \times {\text{ }}T\arg et{\text{ }}INR} \right){\text{ }} - {\text{ }}\left( {0.2538{\text{ }}x{\text{ }}Amiodarone} \right) \hfill \\ \,\, + {\text{ }}\left( {0.0922{\text{ }} \times Smokes} \right){\text{ }} - {\text{ }}\left( {0.0901{\text{ }} \times {\text{ }}African - American{\text{ }}race} \right) \hfill \\ \left. {\,\, + {\text{ }}\left( {0.0664{\text{ }} \times {\text{ }}DVT/PE{\text{ }}as{\text{ }}Indication{\text{ }}for{\text{ }}Therapy} \right) = {\text{ }}estimated{\text{ }}daily{\text{ }}dose} \right] \hfill \\ \end{gathered}$$	$$\begin{gathered} 5.6044 - 0.2614 \times Ageindecades + 0.0087 \times Heightincm \hfill \\ \;\; - 1.6974 \times VKORC1A/A - 0.4854 \times VKORC1genotypeunknown \hfill \\ \;\; - 0.5211 \times CYP2C9*1/*2 - 0.9357 \times CYP2C9*1/*3 \hfill \\ \;\; - 1.0616 \times CYP2C9*2/*2 - 1.9206 \times CYP2C9*2/*3 \hfill \\ \,\; - 2.3312 \times CYP2C9*3/*3 - 0.2188 \times CYP2C9genotypeunknown \hfill \\ \;\; - 0.1092xAsianrace - 0.2760xBlackorAfricanAmerican \hfill \\ \;\; - 0.1032xMis\sin gorMixedrace + 1.1816xEnzymeinducerstatus \hfill \\ \;\; - 0.5503xAmiodaronestatus = Squarerootofweeklywarfarindose \hfill \\ \end{gathered}$$
Additional information	Favoured if smoking status and VTE are known. Easily available at www.warfarindosing.org	Favoured for patients receiving enzyme inducers Could be accessed as an excel sheet in CPIC warfarin guidelines

CPIC: Clinical pharmacogenomics implementation consortium,CYP2C9: Cytochrome 450 2C9, DVT: Deep vein thrombosis, INR: International normalized ratio, MAE: Mean absolute error, PE: Pulmonary embolism, R^2^: Coefficient of determination, VKORC1: Vitamin k epoxide reductase complex 1, VTE: Venous thromboembolism

The next step after derivation and validation of a pharmacogenetic-guided warfarin dosing algorithm is to compare it to the standard of care (either fixed dosing or clinical dosing) in large scale randomized controlled trials (RCTs). Three landmark studies (COAG, EUPACT and GIFT) were published in this essence [38–40]. The trials used a validated genetic guided algorithm (in addition to clinical factors) to predict warfarin maintenance dose in the genetic guided warfarin dosing arm. Most of the population recruited in the three trials were of European descent, except for the COAG trial which recruited more than 25% of patients with African descent. The EUPACT and the GIFT trials showed positive outcomes with respect to genetic guided warfarin dosing when compared to clinical or fixed warfarin dosing. On the other hand, the COAG study did not show difference in the time spent in therapeutic range. Table 4 highlights all the details of these three landmark studies, their key findings and limitations.

**Table 4:** Comparison of the three landmark prospective randomized controlled trials

	EU-PACT 2013	COAG 2013	GIFT 2017
Number of patients.	455	1015	1597
Mean age	67	58	72
Race	98% Caucasians	67% Caucasians	91% Caucasians
Comparator	Fixed dosing	Clinical dosing	Clinical dosing
Genotype guided dosing duration	Five days	Five days	Eleven days
Indications	AF and VTE	AF and VTE	DVT prevention in hip and knee replacement
CYP4F2*3	Not tested	Not tested	tested
Genetic algorithm used	IWPC algorithm	Gage et al. algorithm	Gage et al. algorithm
1ry outcome	PTTR	PTTR	Composite of: major bleeding within 30 days, INR of 4 or greater within 30 days, death within 30 days, and symptomatic or asymptomatic VTE within 60 days of arthroplasty
Result	Genotype-guided dosing was associated with an increased PTTR (adjusted difference = 7%, 67.4% vs. 60% *P* < 0.001).	Genotype-guided dosing was **not** associated with an increased PTTR (adjusted difference= -0.2%, 45.2% vs. 45.4% *P* = 0.91).	Genotype-guided dosing was associated with a lower 1^ry^ composite outcome (absolute difference, 3.9%, 10.8% vs. 14.7%, *P* = 0.02).
Strengths	Study population of same ethnic background	Using Clinical dosing as a comparator (which showed better R^2^ and MAE when compared to fixed dosing)	Large sample size. Tested for CYP4F2*3 in addition to the other genetic variants.
Limitations	Using fixed warfarin dosing as a comparator. (although it could be argued that this may resemble actual practice and increase generalizability of the study)	Included 27% African patients who were tested for genetic variants that were not prevalent in their population. Upon exclusion of the African patients’ data from the analysis, there was a higher PTTR in the genetic guided warfarin dosing (48.8 vs. 46.1%; *P* = 0.15)	Mostly elderly patients. Results of the patients’ genotyping was available before initiation. Composite outcome result mainly driven by the INR > 4 outcome.

AF: Atrial fibrillation, CYP4F2*3: Cytochrome 450 4F2*3, DVT: Deep vein thrombosis, INR: International normalized ratio, PE: Pulmonary embolism, PTTR: Percentage time in therapeutic range, VTE: Venous thromboembolism

## Discussion

Fixed dosing is still a common method to initiate warfarin dosing. Clinical dosing is as an appealing alternative strategy that considers factors proven to affect warfarin dosing when calculating the dose. There has been an indirect comparison between fixed and clinical dosing methods in the IWPC validation study [19]. Clinical dosing showed a higher explanation for warfarin maintenance dose (R^2^ of 26% vs. 0%) and a lower mean absolute error (MAE 9.9 mg vs. 13 mg) when compared to fixed dosing. However, there has been no head to head comparison between fixed and clinical dosing strategies to the best of our knowledge. Genetic dosing that encompasses genetic factors in addition to clinical factors has been studied extensively over the last decade. Many observational studies were able to show that clinical and genetic factors together could explain more than 50% of warfarin maintenance dose variability. However, looking at the bigger picture, three clinical utility studies compared genetic-guided dosing to standard dosing but the results were controversial. Thus, genetic-guided warfarin dosing is still not adopted by guidelines. Because of that reason, the lack of accessibility to genetic testing and the increased use of DOACs, genetic dosing of warfarin is not widely implemented.

## Limitations

While we attempted to include the most relevant studies, the reader is reminded that this is not a systematic review. With regards to fixed dosing method, guidelines were rather outdated (ACCP 2012) and therefore did not reflect the recent evidence in this regards. Also the most robust evidence with regards to genetic-guided warfarin dosing come from the Caucasian population.

## Conclusions

A number of methods exist for warfarin initiation. Studies comparing different dosing methods for initiation yielded conflicting outcomes due to differences in study design, population studied, comparator, and outcomes measured. Looking at the big picture, the use of genetic dosing for warfarin initiation can lead to better outcomes. Whether these better outcomes are clinically or economically beneficial remains controversial. We acknowledge the diminishing role of warfarin in light of the DOACs widespread, but warfarin still remains the first therapeutic option for certain indications and an important therapeutic alternative for others as highlighted in the introduction. Also, the role of pharmacogenetic-guided warfarin dosing could be more obvious in developing countries where patients or insurance systems cannot afford DOACs. Further studies are needed to address these points in light of the decreasing costs and availability of genetic testing.
